# Instructed illiteracy reveals expertise-effects on unconscious processing

**DOI:** 10.3389/fpsyg.2015.00239

**Published:** 2015-03-09

**Authors:** Heiko Reuss, Andrea Kiesel, Carsten Pohl, Wilfried Kunde

**Affiliations:** ^1^Department of Psychology III, Julius-Maximilians-Universität Würzburg, Würzburg, Germany; ^2^Hospital for Psychiatry, Psychotherapy and Psychosomatic Medicine Lohr am Main, Lohr am Main, Germany

**Keywords:** masked priming, expertise, unconscious processing, lexical decision task, top-down control

## Abstract

We used a new methodological approach to investigate whether top-down influences like expertise determine the extent of unconscious processing. This approach does not rely on preexisting differences between experts and novices, but instructs essentially the same task in a way that either addresses a domain of expertise or not. Participants either were instructed to perform a lexical decision task (expert task) or to respond to a combination of single features of word and non-word stimuli (novel task). The stimuli and importantly also the mapping of responses to those stimuli, however, were exactly the same in both groups. We analyzed congruency effects of masked primes depending on the instructed task. Participants performing the expert task responded faster and less error prone when the prime was response congruent rather than incongruent. This effect was significantly reduced in the novel task, and even reversed when excluding identical prime-target pairs. This indicates that the primes in the novel task had an effect on a perceptual level, but were not able to impact on response activation. Overall, these results demonstrate an expertise-based top-down modulation of unconscious processing that cannot be explained by confounds that are otherwise inherent in comparisons between novices and experts.

## INTRODUCTION

The possibilities and limits of unconscious information processing have been an issue of considerable debate (cf. Van den Bussche et al., 2009). Only recently, evidence came up that expertise with a particular stimulus domain is a crucial determinant of the capability to process stimuli that are related to the expertise without awareness. For example, expert chess players (Kiesel et al., 2009) and expert typists (Heinemann et al., 2010) processed unconsciously presented expertise-related information while novices’ performance remained unaffected by the same unconscious stimulation. This also underlines a different (e.g., more configural) processing of expertise-related stimuli, which is a key characteristic of expertise (Gobet and Simon, 1996; Gauthier and Tarr, 1997).

Research on expertise, however, suffers from the notorious methodical problem of relying on a quasi-experimental variable, which invites all kinds of alternative interpretations in terms of subject-related confounds and self-selection problems. In other words, experts may differ from novices not only in terms of practice with a certain task or skill but in other personality traits as well, which eventually determine who becomes an expert and who does not. Ideally, to rule out such subject-related accounts one would wish to study essentially the same subjects once as experts and once as novices.

Here, we suggest such an approach, which we call *de-expertisation*. While all participants responded to the same stimuli in the same way, we allowed only some participants to process stimuli by their expert processing routines, whereas we intentionally *de-expertised* some other participants by instruction.

**FIGURE 1:**
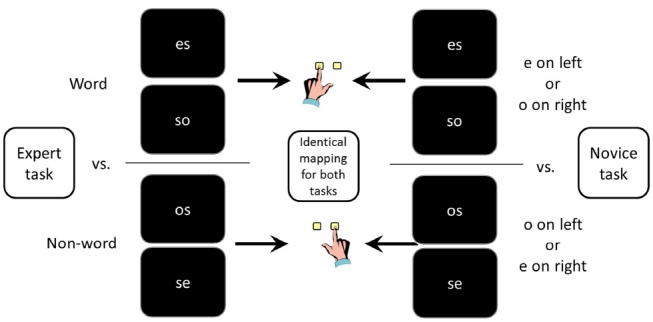
**Stimuli and mapping for both tasks.** All stimuli and the corresponding mapping of stimuli to responses are identical in both tasks. Only the instruction differs: participants are either instructed to respond to the target being a word or a non-word, or to respond to the position and identity of the vowel.

Concretely, half of the participants performed a lexical decision task on the words *es* (German for “it”) and *so* (German for “so”) and the non-words *os* and *se*. This task is based on reading, a skill in which all participants can be considered experts through their long lasting practice (cf. Ericsson, 2006). The other participants responded according to location and identity of the vowel. One response was assigned to an *e* on the left or an *o* on the right side, and the other response to an *o* on the left or an *e* on the right side, resulting in the same stimulus-response mapping as with the lexical task (see Figure 1). Crucially, the expert group addressed a domain of expertise (word reading), whereas the “novice” group performed the task in a way that requires to explicitly combine letter identity and location, which is not associated with any expertise.

Before each target stimulus, a masked prime from the same set of stimuli was presented. This prime either afforded the same response as the target and was thus response congruent (e.g., prime *os* is response congruent to targets *os* and *se*, and prime *so* is response congruent to targets *so* and *es*), or it afforded a different response than the target and was thus response incongruent (e.g., prime *se* is response incongruent to targets *es* and *so*, and prime *es* is response incongruent to targets *os* and *se*). If expertise truly determines whether unconscious stimuli are processed, participants with the lexical task should respond faster and less error prone when the masked prime stimulus is response congruent compared to response incongruent (Dehaene et al., 1998). For the “novices,” however, response congruency of the prime should have no or considerably less impact. In a manner of speaking, the “novices” perform the task in a way in which an illiterate person might approach the task, namely by responding not to the (non-)word status, which would be unknown to them, but by responding simply to perceptual features. Of course, the participants in the novice group were still able to read the words, but when asked after the experiments, none of them indicated to have responded to the words’ status, but indeed to the position of the *o* or the *e* within the target. As such, one might imagine them basically as instructed illiterates.

## MATERIALS AND METHODS

Forty-eight students of the University of Würzburg with an average age of 22 years participated in this experiment, 24 each in the expert group and the “novice” group. All reported having normal or corrected-to-normal vision, were German native speakers and were not familiar with the purpose of the experiment.

The experiment took place in a dimly lit room. An IBM compatible computer with a 17 inch VGA-Display and the software package E-Prime^™^ were used for stimulus presentation and response recording. Stimulus presentation was synchronized with the vertical retraces of a 100-Hz monitor. Responses were executed with the index fingers of both hands and collected with external response keys. At the beginning of each trial, a fixation cross (500 ms) was presented, followed by a forward pattern mask (70 ms), the prime (20 ms), and a backward pattern mask (70 ms). The target was presented directly after the backward mask for 200 ms, followed by a blank screen while waiting for the response. All stimuli were presented in the center of the screen in white Courier New font on black background. The masks consisted of four hash tags and were presented with a point size of 40, prime and target letters were presented with a point size of 36. The target and prime stimuli set consisted solely of the word-stimuli *es* and *so*, and the non-word stimuli *se* and *os*.

The 16 possible prime-target-combinations were used four times in each block, which thus consisted of 64 trials presented in pseudo-random order. After a practice block, participants performed eight blocks with self-paced pauses between each block. At the end of the experiment, we tested prime visibility in a signal detection task. Participants were fully informed about the structure of a trial and the sequence of the presented stimuli. They were instructed to respond to the prime instead of the target. Within an interval of 1000 ms after prime-target presentation participants could not respond (see Vorberg et al., 2003, for implementing such a reversed response window procedure). This was done to avoid measuring the unconscious effect of the prime on the free response choice (see Schlaghecken and Eimer, 2004; Kiesel et al., 2006) instead of the ability to discriminate the prime. To counteract effects of high task difficulty (Pratte and Rouder, 2009), 50% of trials in the prime visibility test contained a visible prime for half of the participants. An experimental session lasted approximately 60 min.

## RESULTS

**FIGURE 2:**
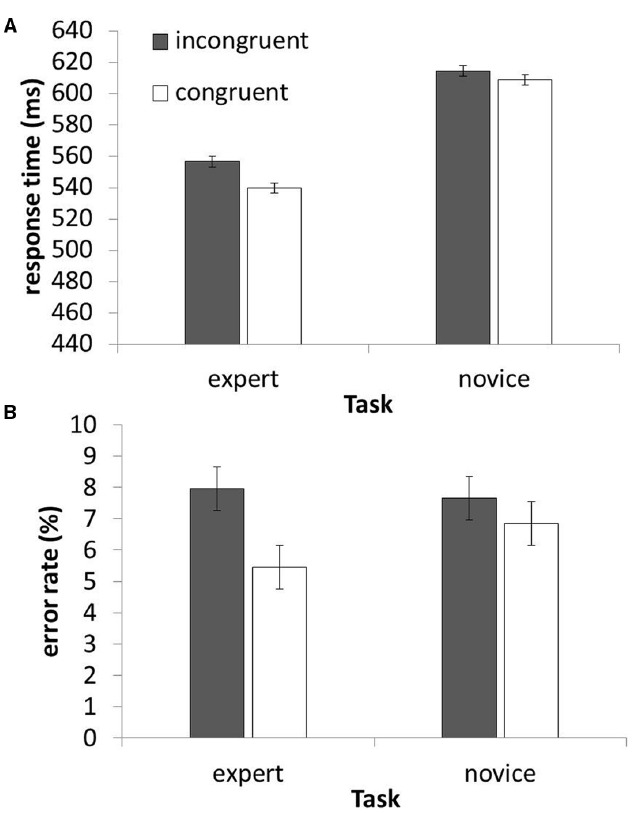
**Results.** RTs **(A)** and error rates **(B)** as a function of task (expert vs. novice) and response congruency. Error bars depict 95% within-subject confidence intervals (Loftus and Masson, 1994).

A mixed-design ANOVA on RTs (excluding error trials and trials with RTs deviating more than 2.5 standard deviations from the participant’s mean RT in the experimental condition of this trial) with the within-subject factor response congruency (congruent vs. incongruent) and the between-subject factor task (expert vs. novice) showed main effects of congruency, *F*(1, 46) = 44.37, *p* < 0.001, and task, *F*(1, 46) = 7.91, *p* = 0.007. Participants responded 11 ms faster for congruent rather than incongruent prime-target pairs, and experts responded 63 ms faster than novices (see Figure 2). Crucially, the interaction of congruency and task was highly significant, *F*(1, 46) = 10.80, *p* = 0.002. This interaction is driven by an 11 ms larger congruency effect in the expert group than in the novice group, *t*(46) = 3.29, *p* = 0.002. Experts responded 17 ms faster after congruent than after incongruent primes, *t*(23) = 6.97, *p* < 0.001, while the congruency effect only amounted to 6 ms in the novice group, *t*(23) = 2.41, *p* = 0.024.

The same ANOVA on error rates revealed a main effect of congruency, *F*(1, 46) = 16.97, *p* < 0.001, with 1.2% less errors with congruent primes, and again an interaction of congruency and task, *F*(1, 46) = 4.46, *p* = 0.040. Experts made 2.5% less errors in congruent trials than in incongruent trials, *t*(23) = 2.64, *p* = 0.023, while errors did not differ significantly between congruent and incongruent trials for novices, *t*(23) = 1.62, *p* = 0.12.

To further elucidate the underlying processes of these results, we excluded trials that featured identical prime-target-pairs. In these trials, response facilitation cannot only be due to response priming, but also due to perceptual priming. By excluding these trials, the remaining congruent trials notably feature no perceptual overlap (neither the location nor the identity of the vowel are the same in prime and target, e.g., *os* and *se*), whereas prime and target in incongruent trials are partially perceptually overlapping (e.g., *os* and *es*). Thus, any response facilitation in congruent compared to incongruent trials indicates processes of response activation that even overcame the perceptual advantage of incongruent over congruent trials. Conversely, faster responses in incongruent trials reflect the impact of a low-level perceptual effect over effects of response activation. The same ANOVA as before on RTs revealed a main effect of task, *F*(1, 46) = 9.267, *p* = 0.004, as well a reverse main effect of congruency, *F*(1, 46) = 4.848, *p* = 0.033, with faster responses after incongruent primes than after congruent primes. Furthermore, we found a highly significant interaction of congruency and task, *F*(1, 46) = 28.630, *p* < 0.001. This interaction is characterized by a regular congruency effect of 6 ms in the expert task, *t*(23) = 2.988, *p* = 0.007, in conjunction with a 14 ms reverse congruency effect in the novice task, *t*(23) = 4.443, *p* < 0.001.

With error rates, again excluding identical prime-target-pairs, this ANOVA reveals a main effect of congruency, *F*(1, 46) = 6.795, *p* = 0.012, which is modulated by an interaction with task, *F*(1, 46) = 5.209, *p* = 0.027. The main effect of task was not significant, *F* < 1. The interaction is characterized by a significant congruency effect (2.0%) in the expert task, *t*(23) = 3.064, *p* = 0.005, with no significant congruency effect (0.1%) in the novice task, *t*(23) = 0.269, *p* = 0.790.

Post-experimental signal detection tests confirmed that there was no difference in prime visibility between the expert and the novice group (*F* < 1), suggesting that any difference in the primes’ effectiveness are not due to differences in prime visibility. In the expert group, *d’* was 0.16, *t*(23) = 2.249, *p* = 0.034, and in the novice group, *d’* was 0.21, *t*(23) = 4.531, *p* < 0.001. To test whether the congruency effect is related to individual prime visibility, a regression analysis as proposed by Draine and Greenwald (1998, see also Greenwald et al., 1995, 1996) was computed. For each participant, a priming index was calculated [100 × (RT incongruent–RT congruent)/RT congruent] as a measure of the primes’ impact on responses. Individual priming indices were then regressed onto individual *d’*-values, task (coded as 0.5 for expert and –0.5 for novice), and the interaction (i.e., product) of both predictor variables. The regression analysis confirmed that task was a significant predictor of priming, *t*(46) = 3.115, *p* = 0.003. In contrast, prime visibility, *t*(46) = 0.164, *p* = 0.870, and the interaction of prime visibility and task, *t*(46) = 0.270, *p* = 0.789, were not significant predictors of priming. A separate analysis for the expert group revealed an intercept that was significantly larger than zero *t*(23) = 6.01, *p* < 0.001. This result implies that a priming effect would still occur with zero prime visibility. Additionally, prime visibility and the priming index were not significantly correlated with each other, *t*(23) = 0.090, *p* = 0.929. Overall, this result pattern (significant intercept, non-significant slope) “is consistent with unconscious cognition dissociated from conscious cognition” (Greenwald et al., 1995, p. 32). In the novice group, there was no significant correlation between prime visibility and the priming index, *t*(23) = 0.292, *p* = 0.773, and the intercept was not significantly larger than zero, *t*(23) = 1.548, *p* = 0.136.

To rule out that the primes remained ineffective for the novices simply because responses were slower overall and the primes’ impact decayed over time, we looked at RT distributions of experts and novices and analyzed whether congruency effects depended on RT level. To this end, RTs of each participant were rank ordered and divided into ten equal-sized speed bins for congruent and incongruent trials. Congruency effects were then calculated for each bin. An ANOVA on congruency effects with the within-subject factor RT bin revealed that there was no main effect of RT bin in the novice group (*F* < 1), suggesting that the level of response speed had no significant influence on the emergence (or non-emergence) of a congruency effect. Furthermore, we analyzed whether the congruency effect in the expert group still differs from the congruency effect in the novice group when overall RT levels are comparable. To this end, we excluded the slowest 20% of congruent and incongruent responses in the novice group, which results in comparable RT levels between the two groups (*F* < 1), before analyzing differences in congruency effects. This comparison still revealed a larger congruency effect in the expert group than in the novice group, *t*(46) = 2.387, *p* = 0.021. This indicates that the observed difference in congruency effects is not caused by overall different RT levels, as a virtual elimination of RT level differences does not lead to an elimination of the differences in the congruency effect.

## DISCUSSION

We investigated whether the effectiveness of masked stimuli can be modulated by instructing participants in a way that either addresses expert processing routines or not. While in terms of stimulus-response-mappings all participants had to perform the same task, the task was described as a lexical decision task for one group of participants, and as a task that requires the combination of several different stimulus features for the other participants. Overall, the results show that masked stimuli impacted on behavior considerably more when participants’ task performance relied on reading expertise, enabling the prime to influence responding by means of word identity. Conceivably, reading enabled holistic processing at the word level (McClelland and Rumelhart, 1981). In contrast, the novice task forced an analytical processing at the letter level, and participants would have to integrate the features identity of the vowel and location of the vowel, which likely requires attention and conscious stimulus representation (Treisman, 1996), thus diminishing the primes’ impact. Furthermore, the congruency effect in the novice task seems to be based solely on low-level perceptual facilitation, but not on response activation, a pattern that can be observed when excluding identical prime-target pairs from the analysis. Here, RTs after response congruent, but perceptually non-identical prime-target pairs were slower than after response incongruent, but partly perceptually overlapping prime-target pairs. This reversed congruency effect thus indicates that the primes were not able to exert an effect based on their response activation (which seems to be absent), but only based on perceptual facilitation.

A similar instance of de-expertisation can be observed when the task-relevance of expertise-related stimuli is manipulated (Harel et al., 2010). When car experts have to recognize car models, the presentation of cars leads to specific brain activity compared to the presentation of planes, a difference that is not found with novices. However, when the task was not to recognize car models, this neural expression of expertise was drastically reduced (i.e., there was no difference between novices and experts). Thus, expertise can indeed be top-down modulated in a way that suppresses even expertise-related activation on a neuronal level (cf. Harel et al., 2013).

The crucial aspect of the design at hand is that the two tasks differ only in their instruction, with everything else in terms of stimuli, responses, and stimulus-response assignments remaining identical. An observer watching a participant would not be able to tell which of the two tasks is performed, as on a basic S-R-level, all participants were doing the same. It is through instructions which either addressed a domain of expertise or not that stimuli are processed differently, but not for example because of changes in S-R-mappings that come along with changing tasks (Norris and Kinoshita, 2008). These results mirror studies that investigated how identical stimuli can be processed differently through top-down or contextual influences (Schyns and Oliva, 1999; Harel and Bentin, 2009; Lupyan et al., 2010; Van Opstal et al., 2011). Of course, it is possible that not only the instructions per se, but also the execution of the differently instructed tasks lead to the differential effects of unconscious stimulation. While instruction itself has been found capable of creating S-R-bindings (e.g., Wenke et al., 2007), it cannot be excluded that task execution played a role in the experiment at hand, too.

One might argue that the two instructions differ not only in addressing expertise or not, but also for example in requiring semantic processing vs. perceptual processing, and thus simply contrast semantic priming with perceptual priming. However, there is no reason to expect an effect of perceptual priming to be absent or smaller than semantic priming. If anything, an effect of semantic priming would be expected to be harder to demonstrate (e.g., Damian, 2001), and both semantic and perceptual priming have been shown to be observable when participants have according action goals (Martens et al., 2011). Thus, the observed result pattern is unlikely caused simply by semantic and perceptual priming leading to different (or absent) priming effects. Likewise, the results could be seen as a consequence of holistic vs. analytical processing. This does not constitute an alternative explanation compared to an effect of expertise, but holistic processing is in fact a central aspect of expertise, which has for example been demonstrated numerous times with expert chess players (de Groot and Gobet, 1996; Gobet and Charness, 2006). Holistic processing of the prime stimulus, triggered by addressing expert processing routines, was conceivably a central mechanism that enabled an impact of the unconsciously presented prime.

Regarding general mechanisms of unconscious processing, our findings confirm the assumption that the processing of unconsciously presented stimuli hinges on suitable top-down settings (e.g., Kunde et al., 2003, 2005; Kiefer and Brendel, 2006; Kiefer and Martens, 2010; Martens et al., 2011). Martens and colleagues showed that when a preceding task induces a particular task set (e.g., a semantic vs. a perceptual task set), the effect of masked primes is modulated by this task set. For example, after a semantic task, the primes’ impact was based on their semantic value, while after a perceptual task, semantic value of the prime had no impact. Likewise, in the study at hand, different top-down settings were induced by different instructions for technically the same task, which strongly modulated the way primes were processed.

To conclude, we showed that expertise crucially influences the possibilities of unconscious processing. In contrast to earlier studies on this subject, we did not compare experts and novices, but varied task instructions so that expertise in reading was either addressed or not. The observed effects therefore cannot be attributed to ulterior differences between groups, but are unambiguously linked to expert processing routines.

### Conflict of Interest Statement

The authors declare that the research was conducted in the absence of any commercial or financial relationships that could be construed as a potential conflict of interest.
